# Emergency department patient safety incident characterization: an observational analysis of the findings of a standardized peer review process

**DOI:** 10.1186/1471-227X-14-20

**Published:** 2014-08-08

**Authors:** Zach K Jepson, Chad E Darling, Kevin A Kotkowski, Steven B Bird, Michael W Arce, Gregory A Volturo, Martin A Reznek

**Affiliations:** 1 Department of Emergency Medicine, University of Massachusetts Medical School, 55 Lake Avenue North, Worcester, MA 01655, USA

**Keywords:** Patient safety incidents, Peer review, Emergency department

## Abstract

**Background:**

Emergency Department (ED) care has been reported to be prone to patient safety incidents (PSIs). Improving our understanding of PSIs is essential to prevent them. A standardized, peer review process was implemented to identify and analyze ED PSIs. The primary objective of this investigation was to characterize ED PSIs identified by the peer review process. A secondary objective was to characterize PSIs that led to patient harm. In addition, we sought to provide a detailed description of the peer review process for others to consider as they conduct their own quality improvement initiatives.

**Methods:**

An observational study was conducted in a large, urban, tertiary-care ED. Over a two-year period, all ED incident reports were investigated via a standardized, peer review process. PSIs were identified and analyzed for contributing factors including systems failures and practitioner-based errors. The classification system for factors contributing to PSIs was developed based on systems previously reported in the emergency medicine literature as well as the investigators’ experience in quality improvement and peer review. All cases in which a PSI was discovered were further adjudicated to determine if patient harm resulted.

**Results:**

In 24 months, 469 cases were investigated, identifying 152 PSIs. In total, 188 systems failures and 96 practitioner-based errors were found to have contributed to the PSIs. In twelve cases, patient harm was determined to have resulted from PSIs. Systems failures were identified in eleven of the twelve cases in which a PSI resulted in patient harm.

**Conclusion:**

Systems failures were almost twice as likely as practitioner-based errors to contribute to PSIs, and systems failures were present in the majority of cases resulting in patient harm. To effectively reduce PSIs, ED quality improvement initiatives should focus on systems failure reduction.

## Background

It has been stated that “medicine used to be simple, ineffective, and relatively safe; now it is complex, effective, and dangerous” [1], confronting us with the notion that modern health care delivery is error prone. As early as the turn of the previous century, patient safety pioneers began to understand this [2,3], but it was not until the publication of the landmark Institute of Medicine (IOM) report, “To Err is Human” [4], in 1999 that healthcare communities in general began to understand the magnitude and gravity of “errors” in healthcare. Since the IOM report, organizations such as the World Health Organization have recommended using the term ‘patient safety incident’ (PSI) as opposed to ‘medical error’ [5], but the principles remain unchanged. A PSI has been defined as any unintended or unexpected incident that could have or did lead to the harm of a patient [5].

“To Err is Human” demonstrated that our current health care system is flawed and often puts our patients at risk of harm. This may hold particularly true for care in the Emergency Department (ED) which has been identified as a patient care setting that is prone to PSIs for a variety of reasons including a chaotic work environment, high patient acuity, multiple transitions in care, and ED crowding [6-8]. Some reports have suggested that PSIs in emergency medicine (EM) occur in diagnosis, pharmacotherapy, procedures, and communication [8-12], and that many ED PSIs may be preventable [12]. Despite the clear importance of this area of research, studies describing ED PSIs remain limited in number and vary greatly in their scope and methodology [10-13]. Consequently our understanding of PSIs in EM remains incomplete to date, and further studies are needed to better characterize PSIs that occur in the ED setting as well as circumstances in which they ultimately result in patient harm. Peer review has been cited as one potentially valuable source for PSI identification and analysis [13,14].

The primary objective of this investigation was to identify, analyze and characterize ED PSIs via a standardized, peer review process. Secondary objectives included: 1) the analysis and characterization of circumstances in which PSIs led to patient harm and 2) to describe in detail the standardized, peer review process itself so that others may use it as a template for similar quality and safety improvement processes in their institutions.

## Methods

### Study design, setting, and population

This was an observational study conducted at a tertiary care, adult, urban ED with a university affiliation and an approximate annual census of 66,000 patients from October 2010 through September 2012. The site is an American College of Surgeons-verified Level I trauma center and also serves as a regional stroke and ST-elevation myocardial infarction referral center. The ED is the primary teaching site for an accredited 3-year EM residency. Board certified EM physicians care directly for the majority of patients either as the sole provider or through direct supervision of EM and rotating residents. Approximately 5% of patients are seen primarily by mid-level practitioners; an attending EM physician may or may not be directly involved in the care of those patients but is directly available for supervision as needed. The data collection and study methods were reviewed and approved by the University of Massachusetts Medical School Institutional Review Board.

### Methods and measurements

**Figure 1:**
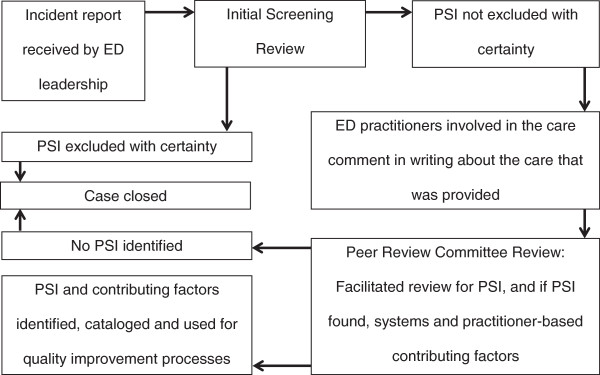
Flow diagram of the peer review process.

In order to improve the identification and analysis of potential PSIs in the ED, a highly structured peer-review process was implemented in 2010 (Figure 1). The format of the peer-review process was based on one previously described at Detroit Receiving Hospital (DRH) [13]. All ED incident reports were analyzed via the peer review process. Incident reports were submitted via a standardized, electronic, hospital incident reporting process or as direct verbal, written or electronic communications to ED leadership. Incident reports originated from both clinical and non-clinical individuals and groups including but not limited to: patients, patients’ families, ED providers (nurses, residents, attending physicians), inpatient providers and consultants, hospital quality and clinical care committees, and providers from outside the hospital. In order to promote reporting from hospital-based sources, practitioners and committees were informed and reminded about the ED peer-review system on an ongoing basis.

Every incident report submitted to the attention of ED leadership was analyzed via the peer review process as a matter of policy. For practical considerations, each incident report first underwent a preliminary screening review by the ED Clinical Director/Vice-Chairman of Clinical Operations to determine if there was any potential for a PSI having occurred. If it could be determined with “absolute certainty” that no PSI had occurred, the case was excluded from further review. (For example, the perceptions of the incident reporter were determined to be factually inaccurate.) Otherwise, the default would be for the case to progress to a full Peer Review Committee (PRC) evaluation. The PRC was open to all physicians, residents and mid-level practitioners in the ED. A core group of eight board-certified emergency physicians attended the monthly PRC meetings regularly to ensure accuracy and consistency in the review process.

Prior to the PRC evaluation of an incident report under review, all ED practitioners involved in the case received a peer review response packet which included a copy of the relevant portions of the patient’s medical record and a peer review response document with prompting questions related to the quality concerns in the case. The prompting questions were specifically tailored to each case, but in general each had a broad prompting question or statement such as “please comment on the care provided” or “did you perceive any opportunities for improvement in the care?” Other more specific questions were included as necessary to ensure response to a specific area of concern suspected by the reporter or preliminary reviewer, for example, “did you perceive a delay in diagnosis, and if so could you please comment on potential contributing factors?” Each practitioner was required to return the peer review response document after commenting in writing about the care that was provided and any other circumstances related to the specific quality or safety issues in question. This allowed for information pertinent to the case that might not be available in the medical record to be presented to the PRC committee during the case review. The provider(s) involved in the case were also encouraged, but not required, to attend the PRC meeting. At the PRC meeting, all committee members reviewed the medical records and the practitioners’ written feedback. The medical records (and the practitioners’ written feedback if they did not attend the PRC review) were de-identified.

Following review of the medical records and response documents, each case underwent a period of facilitated discussion related to potential PSIs. Specifically, the committee reviewed each case for six types of systems failures and five types of practitioner-based errors (Table 1). The classification system utilized in the peer review process was developed based on systems previously reported in the emergency medicine literature as well as the investigators’ experience in quality improvement and peer review. The practitioner-based error classification was modeled after a system reported by Berk et al. [13]. For systems failures, the classification approach was based on portions of a framework suggested by Cosby [15]. Generally, there was group consensus when systems failures were identified; however, if there was any dispute, a majority vote determined whether systems failures had occurred. For practitioner-based error identification, a majority vote determined whether one had occurred. Average committee attendance was ten per meeting of which board-certified and board-eligible attending physicians accounted for 86%. All committee participants were eligible to vote in general, however providers involved in a case in question were required to leave the room for the vote on practitioner-based error in that specific case in order to reduce influence on the committee determination.

**Table 1:** Classification criteria for systems failures and practitioner-based errors identified by the PRC

**Type of PSI**	**Definition**	**Example**
**Systems failures**		
Triage	A failure in assessment of potential disease severity during triage	Abnormal vital signs not recognized as a potential sign of shock
ED teamwork	A failure due to an issue with ED staff communication or a shared responsibility across multiple ED staff	Change in vital signs not communicated to the attending physician
Hospital Teamwork	A failure due to an issue with communication between ED and hospital staff or a shared responsibility between the ED and hospital staff	Pertinent information not communicated to the admitting team
ED work environment	A failure resulting from the lack, malfunction, or mal-design of resources, equipment, or physical space within the ED or a failure due to not following an ED policy or clinical practice guideline	Missing equipment
Hospital work environment	A failure resulting from the lack, malfunction, or mal-design of resources, equipment, or physical plant outside the ED but still within the hospital or a failure due to not following a hospital policy or clinical practice guideline	Specialty testing areas remotely located from the ED
Boarded patient	A failure occurring after a patient is admitted to an in-patient service but is still physically located in the ED	N/A
**Practitioner-based errors**		
Major cognitive error	An error which represents serious mismanagement in a knowledge area basic to EM	Failure to diagnose or treat ST-elevation myocardial infarction
Cognitive error	An error which represents mismanagement which is either less serious than a major cognitive error or in an area less basic to EM	Failure to consider the institutional antibiogram during antibiotic selection for treatment of simple urinary tract infection
Missed radiographic finding	An error in interpretation of a radiographic study that did not reach the level of a cognitive or major cognitive error	Missed fracture on radiographic interpretation that was splinted correctly based on clinical suspicion
Policy deviation	An error in following a clinical or administrative policy, guideline or standard practice that does not reach the level of cognitive or major cognitive error	Failure to alert the transplant service when a transplant patient is in the ED
Procedural error	A technical error during performance of a procedure that does not reach the level of a cognitive or major cognitive error	Insufficient sterile technique

Finally, the PRC analyzed each case in which one or more systems failures or practitioner-based errors were identified to determine if the PSI resulted in patient harm. Harm was classified as temporary, permanent or death. If it could not be determined definitively that the PSI directly caused or contributed to patient injury or death, patient harm was classified to be “no harm or unknown”.

The entire peer review process, including the cataloging of cases and the practitioner response documents, was executed in a fashion that complied with state legal statutes related to peer review protection. For information to be protected from legal discovery, the following requirements were followed: all written and electronic documents were titled “confidential: peer review”, all documents were stored in locked files and/or password protected databases and verbal discussion were not allowed outside of specifically designated peer review meetings. As a final protective measure, if any written or electronic communication was required related to the peer review findings, the communication did not contain any patient or provider identifiers.

### Data collection and analysis

The primary outcomes reported in this study were the types and frequencies of systems failures and practitioner-based errors contributing to PSIs identified by the peer review process over a 24-month data collection period. PSIs and contributing factors identified by the peer review process were cataloged prospectively in a quality assurance database. This database was reviewed and descriptive statistics were calculated. In addition, retrospective chart review of each case in which a PSI was identified was performed to determine patient age, sex, and primary ED diagnosis. Primary ED diagnoses were classified into twenty systems/disease-based diagnostic categories: cardiovascular, dental, dermatologic, endocrine, gastrointestinal, hematologic, infectious disease, musculoskeletal (non-trauma), neurologic, obstetrical/gynecologic, oncologic, ophthalmologic, otolaryngologic, psychiatric, pulmonary, renal, toxicologic, trauma, urologic, and unknown.

The secondary outcomes reported in this study were the types and frequencies of systems failures and practitioner-based errors that occurred in cases in which the peer review process determined that patient harm definitively resulted from a PSI. Incidents of patient harm resulting from PSIs were cataloged prospectively in the peer review database. The database was reviewed and descriptive statistics were calculated.

## Results

Over a period of 24 months, 469 incident reports were analyzed via the peer review process. Of the 469 cases, 188 (40%) met criteria for further analysis by the PRC and 281 (60%) were found to have no PSIs during the initial screening process. Of the 188 cases reviewed by the PRC, 152 were found to have one or more systems failures or practitioner-based errors. In total, 188 systems failures and 96 practitioner-based errors were identified in the 152 cases. The most common systems failures involved teamwork, and the most common practitioner-based errors were classified as cognitive errors (Table 2).

**Table 2:** Systems failures and practitioner-based errors identified by the peer review process

**Systems failures (n = 188)**	**N (%)**	**Practitioner-based errors (n = 96)**	**N (%)**
ED teamwork failures	79 (42)	Cognitive errors	65 (68)
Hospital teamwork failures	59 (31)	Major cognitive errors	24 (25)
Boarded patients	26 (14)	Missed radiographic findings	4 (4)
ED work environment failures	14 (7)	Policy deviations	3 (3)
Hospital work environment failures	6 (3)	Procedural errors	0 (0)
Triage failures	4 (2)		

The mean and median age of the patients in whose care a PSI was identified were 53.7 and 56 years, respectively, and 49.0% were female. The most frequent diagnostic categories for cases in which a PSI was identified were: infectious disease (15.8%), neurologic (13.2%), cardiovascular (12.5%), gastrointestinal (11.2%), trauma-related (10.5%), non-trauma related musculoskeletal (7.9%) and urologic (5.3%). The remaining diagnostic categories each included less than 4.0% of the cases in which a PSI was identified.

In 140 of the 152 cases (92%) in which PSIs occurred, the PRC determined that no or unknown harm resulted from the PSIs. In twelve cases (8%), the PRC determined that patient harm definitively resulted (Table 3). Of these twelve cases, eleven (92%) involved one or more systems failures, and nine (75%) involved a practitioner-based error. Eight of the nine cases of harm with a practitioner-based error (89%) had a concomitant systems failure, and only in one case of the twelve (8%) did a practitioner-based error alone lead to patient harm. In ten of the twelve cases (83%) of PSIs resulting in patient harm, multiple failures/errors were determined to have occurred, and multiple failures/errors were determined to have occurred in all three of the most serious cases of resultant harm (permanent harm or death). ED teamwork failures and major cognitive errors occurred in each of the cases of permanent harm or death, and in two of these cases, hospital teamwork failures also occurred.

**Table 3:** Systems failures and practitioner-based errors identified in cases of patient harm

**Case**	**Patient harm**	**System failures and practitioner-based errors contributing to harm**
1	Death	ED teamwork, major cognitive error
2	Death	ED teamwork, hospital teamwork, boarded patient, major cognitive error
3	Permanent harm	ED teamwork, hospital teamwork, major cognitive error
4	Temporary harm	ED teamwork, cognitive error
5	Temporary harm	ED teamwork, hospital teamwork, cognitive error
6	Temporary harm	ED work environment, cognitive error
7	Temporary harm	Cognitive error
8	Temporary harm	ED teamwork, hospital teamwork, major cognitive error
9	Temporary harm	ED teamwork, boarded patient
10	Temporary harm	ED teamwork, hospital teamwork, major cognitive error
11	Temporary harm	ED teamwork, Hospital work environment
12	Temporary harm	Hospital teamwork

## Discussion

The results of this investigation demonstrate that PSIs occur frequently in the ED. Contributing to the identified PSIs, systems failures were almost twice as common as practitioner-based errors. Furthermore, systems failures occurred in over 90% of the cases in which patient harm was determined to have resulted from the PSI. Only in one case did harm result from a practitioner-based error in isolation. These findings suggest that systems failures within the ED work environment contribute more significantly to PSIs and patient harm.

To date, investigations of PSIs in the ED setting have been fairly limited in number despite the importance and urgency of improving our understanding of PSIs. Contributing to this relative lack of data is the fact that no clear consensus on optimal methodologies in this area of research exists. Of the few studies that do exist in EM including this one, all have highly disparate methodologies [8,9,11-13]. Because of their varied study designs, the ED PSI investigations have unique potential advantage and disadvantage profiles. For example, the National Emergency Department Safety Study was a large multi-center study that identified PSIs by structured chart review but only focused on a limited number of medical conditions: myocardial infarction, asthma exacerbation, and joint dislocation involving procedural sedation [8,12]. Another study by Fordyce et al. prospectively detected PSIs through intense direct observation of ED providers and staff, however, the study was limited to a period of one week [11]. Smith et al. attempted to identify PSIs in a focused area, transitions of care in the ED, by querying ED residents about their perceptions of error [9]. Finally, the only peer review-based investigation other than the present study, Berk et al., focused on practitioner-based errors and did not report on systems failures [13].

In contrast to the aforementioned studies, the unique strengths of the present study design included a structured, non-punitive peer review process that incorporated feedback from the practitioner(s) involved in the care in question, allowing for first-person accounts of the case under review. Additionally, case reviews were performed by a committee that included multiple practicing, board certified, EM physicians which we believe strengthened the committee’s ability to determine the presence or absence of PSIs. Lastly, the present study examined potential PSIs occurring over an extended two-year time frame and was not limited by diagnosis-based inclusion criteria.

Given this unique design advantage profile (as well as some potential limitations discussed below), the present investigation complements the existing, limited body of original investigations of ED PSIs. Our results are consistent with those prior studies that have implicated work systems as being intimately involved in ED PSIs. For instance, other investigators have stated that ED systems must be changed in order to lower the incidence of errors [11], and Camargo et al. recently concluded that systems factors such as staffing, teamwork, and safety culture are important mediators of error in the ED [12]. In aggregate, ED PSI studies, including the present investigation, provide a significant, growing body of evidence supporting those who have argued that heightened focus should be placed on the work environment and other factors that contribute to error rather than on the error itself [14,16,17].

A unique finding of the present study design that warrants further discussion is the fact that nearly 60% of the incident reports were found to have no potential for a PSI on initial screening. Anecdotally, a few of these reports were submitted by healthcare providers that were not aware of all of the facts of a case at the time that they submitted their incident report, but the majority of the reports that did not progress past the initial screening involved complaints submitted by patients and families. Data from previous investigations have suggested that patient and family concerns often are related to incomplete or delayed relief in symptoms, suboptimal practitioner communication, or billing related to their medical services [6,18,19]. Our anecdotal experience matched these previous reports. While such feedback was still highly valued for its potential to improve the overall patient experience (and was acted upon via other mechanisms within the department), it did not meet criteria to progress to peer review committee analysis because a safety incident had not occurred.

### Limitations

The present study has several design strengths including the highly structured peer review process and the extended study time-period, however, it also has potential limitations that must be considered when interpreting the results. First, any peer review process necessarily requires human interpretation and as such may be prone to bias [17,20,21]. Several measures were designed into the peer review process to reduce the potential for bias, but it was unlikely that all bias was eliminated. A second potential limitation was the fact that the preliminary screening was performed by a single reviewer. While not ideal, this format was necessary due to practical considerations. To minimize potential accuracy or bias limitations inherent in single-reviewer screening, the chosen preliminary reviewer had greater than ten years of professional experience in peer review as well as health-care quality management in general. In addition, the preliminary review process was designed to be more inclusive to minimize the potential for missing any true PSIs. It is not possible to determine definitively if this approach was effective in this regard, however the finding that 36 cases (19.1%) that did meet initial screening criteria subsequently were found not to have PSIs on full review suggests that the approach was at least in part effective. A third potential limitation of the present study was that the review process only identified harm having occurred if a causal relationship between a PSI and patient harm was definitive. This likely increased the false negative rate for harm causality.

A final potential criticism of the present study design could be that it may have missed a significant number of PSIs because under-capture of PSIs is known to occur with passive incident reporting systems in inpatient settings [22-24]. Theoretically, this phenomenon likely also exists in the ED setting, but the extent to which it occurs in the ED has yet to be studied objectively. Some have suggested automatic reviews of 72-hour ED returns, deaths within a certain time frame of admission from the ED, and ED deaths as additional potential sources for improved PSI capture in the ED [13,25]. However, the effectiveness of these methods have not been studied, so the extent to which the results may have been affected by not including them in the study methodology is unclear. Of note, ED deaths were automatically reviewed via a separate process within the institution. Over the two-year period of this study, this separate ED death review process found only two cases having concerns for PSIs, and both in fact were captured independently by the peer review process described in this investigation. This may provide some support toward automatic ED death reviews not being of additional benefit. Also of note, the capture rate of practitioner-based errors in the present study were similar to those reported in a prior study that did include automatic review of 72-hour ED returns resulting in admission, deaths in the ED, and deaths within 24 hours of admission from the ED (systems failures were not reported in that study so it was not possible to compare the system failure capture rates) [13]. It therefore also remains unclear if adding automatic reviews of 72-hour returns or deaths within 24 hours of admission would have increased the observed PSI capture rate in this study. We are aware of no specific data to support the following hypothesis, but it may be possible that the phenomenon of under-reporting of incidents may not be as pronounced in the ED as other healthcare settings. Nearly all patient care occurring in the ED results in a hand-off to an inpatient or outpatient team. Those teams may be more likely to report incidents both because PSIs may become more apparent with the passage of time and because there may be fewer perceived disincentives to reporting if the reporter or their departmental colleagues were not primarily responsible for the care in question. While it is not possible to determine the extent of under-reporting of incidents that occurred during this study, for the reasons outlined above, we believe that the effect may not have been as significant as some may theorize. In addition, while under-reporting may have resulted in the total number of identified PSIs being low, we have no reason to suspect that the phenomenon would bias the proportion of systems failures versus practitioner based-errors, although we acknowledge that it remains possible.

## Conclusions

The results of this investigation reveal that systems failures lead to PSIs and patient harm more frequently than practitioner-based errors in the ED. These findings suggest that to effectively reduce PSIs and patient harm, systems failure prevention should be a priority within ED quality programs.

## Competing interests

The authors declare that they have no competing interests.

## Authors’ contributions

MAR and GAV conceived of this study. ZKJ, MAR, CED, MWA contributed to the study design. ZKJ, MAR performed the data collection and analysis. CED, SBB provided statistical advice. ZKJ and MAR drafted the manuscript, and all authors contributed substantially to its content and finalization. All authors read and approved the final manuscript.

## Authors’ information

MAR was the Vice-Chairman for Clinical Affairs in the Department of Emergency Medicine. He has also served previously as a Vice President of Quality and Patient Safety in a large, urban, teaching hospital. KAK was the Fellow in Emergency Medicine Administration and Leadership. MWA and ZKJ were residents in the Department with interests in Emergency Medicine administration. SBB was Vice-Chairman of Academic Affairs and the Residency Program Director. GAV was the Chairman of the Department of Emergency Medicine. CED had extensive original research publication experience in multiple areas of Emergency Medicine and an interest in quality improvement.

## Pre-publication history

The pre-publication history for this paper can be accessed here:

http://www.biomedcentral.com/1471-227X/14/20/prepub
